# Visualising difference, similarity and belonging in paediatric genetics

**DOI:** 10.1111/j.1467-9566.2011.01388.x

**Published:** 2011-11-03

**Authors:** Janice McLaughlin, Emma K Clavering

**Affiliations:** School of Geography, Politics and Sociology, Newcastle University

**Keywords:** paediatric genetics, family, childhood, disability

## Abstract

Paediatric genetics is increasingly playing a role in explorations of why a child may not be reaching developmental milestones, while experiencing various health concerns and displaying unusual physical characteristics. The diagnostic processes include close analyses of a child’s body in order to identify ‘clues’ to possible genetic variation. When the genetic variation identified is new and complex there is significant uncertainty about what relationship that variation has to childhood development and what it will mean for a child’s future. This paper, drawing from an ethnographic study of a genetics clinic, explores what versions of childhood difference and normality are produced by genetic explorations marked by uncertainty. The focus is on the significance of visual dynamics within the consultation, in family stories or photographs, and in the images found on websites which catalogue genetic syndromes. Our argument is that inside and outside the clinic the visual interpretations create understandings of the child that at times position him or her as ‘other’, while at other times recognise the child as normal and ‘one of us’. The uncertainty embedded in identifying rare genetic variations enables multiple interpretations to emerge which do not ‘fix’ the child into the category of the ‘genetically other’.

## Introduction

Children can now be tested for a range of single gene disorders that have established pathways to diagnosis and treatment, for example Phenylketonuria, Achondroplasia and Cystic Fibrosis. However, paediatric genetics also explores less established associations between variations within (deletions) and across (translocations) chromosomes and problems in childhood development. Some of the associations have become established enough to be given a name, others are so unique they are only referred to via the pattern of chromosomal difference found (for example, ‘2q37 deletions’). Whether named or not, such associations, for now at least, are often unable to generate clarity in terms of both prognosis and treatment. In the future the range of associations that can be drawn between genetic variation and development differences is likely to grow. One reason for this is the increased use of DNA microarray analysis within both genetic research and clinical practice (Miller *et al.* 2010). For example, a number of international research projects are underway using microarray analysis to explore the role genetics may play in autistic spectrum disorder (Pinto *et al.* 2010, Shen *et al.* 2010).

The history of medicine contains many diagnostic processes, which over time become routinised and associated with treatment avenues. It can be argued that even while the clinical benefit of new diagnostic approaches remains disputed, in the meantime what they can provide is social utility. What utility is provided by a diagnosis is contingent on the social, political, economic and cultural location of children and their families: for example, the healthcare and social care system they are reliant on; the resources or capital they hold; and the meanings they derive from medical diagnosis (Rapp and Ginsberg forthcoming). The counterargument from within disability studies is that – whatever the social utility such diagnoses and research provide – they produce a growing medicalisation of human variation (Kerr and Shakespeare 2002). For example, an important concern is whether the position of the child within the family is undermined by focusing on what is different about him or her (Finkler 2001, Asch 2006). We may ask then, in a context where clinical utility is limited, what kinds of framings of childhood difference are produced within the processes of genetic diagnosis? Furthermore, who are the important actors producing such framings, and how do they establish potentially competing framings over and above others?

This paper explores these questions via discussion of an ethnographic study of families accessing a genetics centre in the UK. The focus of the study is children who are referred to the centre because they have development problems and/or unusual physical characteristics which other medical investigations have been unable to explain. The diagnostic process begins with dysmorphology (the visual analysis of the child’s body aimed at identifying ‘abnormal’ features) and explorations of family history, before then moving on – over time in a process geneticists refer to as ‘watchful waiting’– to also involve various analyses of the child’s (and sometimes parents’) blood. The study is examining a range of interrelated issues, for example what the production or non-production of a genetic explanation means for families (McLaughlin and Goodley 2008); whether it makes a difference that a genetic syndrome is inherited or new; different understandings of social and biological inheritance; the production of moral tales of family boundaries of inclusion and exclusion; different practices of authority within the diagnostic process; and children’s own understandings of family and genetics (Clavering and McLaughlin 2010). What the different aspects share is a focus on the interplay of understandings of genetics, family, and childhood and the significance of such interplay for questions of identity and selfhood.

In this paper we focus on the understandings of childhood difference brought into social and medical significance through visual aspects of the diagnostic process. Within this we are looking at several things: first, the form of medical gaze produced in the examination of the child’s body; second, the interpretation of visual representations of the child and other children with similar patterns of genetic variation; and third, how representations and understandings of family and kinship influence the interpretation of the child’s body. Before examining these three aspects of the diagnostic process we provide some context to the paper, first, by highlighting existing literature about the production of childhood otherness, and second, by describing our project.

## Childhood otherness

When a young child is not reaching important developmental markers it ‘disrupts taken-for-granted assumptions about the mind and body’ (Kelly 2005b: 181), raising questions for what future she or he may have. When this is combined with physical features which mean that a child looks different to others then the child can fall under a social gaze which frames him or her as ‘damaged’ (Lindemann Nelson 2001), ‘monstrous’ (Shildrick 2002), or ‘stranger’ (Hughes 2002). Lindemann Nelson uses the notion of damage to signify the ways in which problematic social understandings of people who are thought of as different ‘damage’ the person by imposing an identity onto them. This imposed identity, for example presenting a child or adult who is disabled as someone who is tragic and to be pitied, creates misrecognition of who they are or can be, and limits the space they have to define for themselves who they are, can be or indeed want to be. In this context of social othering, one role medicine can play is to reduce the socially noticeable differences associated with a child’s disability (McLaughlin 2005, McLaughlin and Goodley 2008). For example, drugs to minimise the disruptive behaviour associated with ASD, or, surgery to minimise the distinctive physical characteristics associated with Down’s syndrome or restricted growth (Hansen and Hansen 2006, Parens 2006). Part of the rationale behind parents’ actions can be understood as seeking to shield the child from the social costs of difference (Rehm and Bradley 2005, Kittay 2006).

The danger however, from the perspective of disability studies (Molloy and Vasil 2002), is that rather than being a solution to the problem of explaining childhood difference, medicine can become part of the problem (Conrad and Potter 2000, Gillman *et al.* 2000, Blum 2007). Historically the association of disability with tragedy is a damaging discourse which medicine helped produce (Barnes and Mercer 2003). Once a child is provided with a medical category, even one sought with the goal of providing support and treatment, it becomes difficult to escape that category and for the child to be read independently of it (Landsman 2003, Kelly 2005a). In this way difference is produced and secured in the attempt to explain difference. In relation to paediatric genetics, Featherstone *et al.* (2006: 101) query whether ‘the diagnosis of a genetic condition can place in hazard the identity of a child’. Shaw *et al.* (2003) focus on dysmorphology’s link back to nineteenth century phrenology – the categorisation of population types via ‘unusual’ physical characteristics – in order to argue that this history creates an association with medicine’s complicity in the production of stigmatised, monstrous identities/populations (Paul 1998).

However, it is important to pause and place paediatric genetics within its social and cultural context, in particular, to recognise the multiple ways in which categories of the ‘acceptable person’ are produced (Landsman 2003, Carsten 2004, Kittay 2006, Raspberry and Skinner 2007, Fitzgerald 2008). While medicine can be a factor in dynamics of stigmatisation and othering, this does not occur in a context-free vacuum. Of at least equal significance are the social and cultural notions of shame and exclusion which operate at the level of kinship and community (Gray 2002). Kinship dynamics of difference and othering are an important aspect to bring into the analysis of paediatric genetics, not least because the diagnostic process brings family history and therefore family narratives of belonging and difference into the foreground. Family is hugely significant in shaping the identity of children, within complex social and cultural productions of kinship connection and disconnection (Carsten 2004). Anthropological insight highlights the importance of rhetorics of shared biological connection – which can now be articulated as the shared substance of DNA – in social and cultural framings of legitimate kinship formations (Schneider 1968, Carsten 2004). Strathern (1992: 53) argues that genetics has become a cultural tool in contemporary constructions of the substance of kinship, emphasising ‘the naturalness of biological kinship’. Children themselves are material and embodied versions of the substance of kinship relations. This is why when children come into a family through different routes, such as with the aid of reproductive technologies, or adoption, or through the remaking of family ties via step-families, families work to both position the child as one of them, and develop practices of family which provide their formation with social legitimacy (Haimes 2003). If a child is thought of as different and other, this can put in jeopardy well sculpted understandings of the social legitimacy of family ties. For example, questions can be raised about whether poor parenting has contributed to their child/ren’s problems; or whether the mother did something damaging such as smoke or drink during pregnancy. Therefore, a relationship exists between how a child who appears to be different is framed and the validity of kinship formation.

What is required is an examination of how genetic investigations and representations and ongoing practices of kinship come together to frame the significance of differences in a child’s development and physical features.

## Methodology

This article is based on data emerging from ongoing fieldwork with 17 families of children newly referred to a genetic service in the UK.^1^ The service is based within one hospital trust, but undertakes clinics across a large rural and urban region. Referrals, usually triggered by paediatricians looking after the child, lead to an initial consultation at one of the clinics. All first (and subsequent) consultations are led by a geneticist, although others, usually a paediatrician or a medical trainee, can sometimes be present. If the geneticist feels that there is little indication from the consultation that further genetic exploration could provide anything of value (usually framed as to the family), then things can end there. If, instead, the geneticist thinks there is something they can look for, then further consultations would occur to establish whether a particular syndrome or pattern of polygenetic variation can be found, and whether this could be explained via inheritance from one or both parents or a new mutation.

The study is based in an area of England associated with significant socio-economic deprivation, and a predominately fairly stable White, working class population, with continued patterns of strong kinship ties. This background is reflected in the lives of the families – the majority (N = 14) defined themselves as working class, several (N = 7) were located in dispersed ex-mining villages or semi-rural locations across the region, while all families lived close to or had strong connections to extended kin. Our methodological approach follows each family over time (up to 18 months from first referral), going with them into the different settings of their lives and listening to the perspectives of multiple actors within the family, including parents, siblings, other significant family members and the children marked as different themselves. Fieldwork data is generated through a mix of qualitative longitudinal interviews and non-participant observation in clinical and non clinical encounters. Recruitment occurred through letters of invitation sent via the genetics service. We have put in place a number of measures, as part of the overall approach towards protecting the anonymity of the participants, to ensure that the clinic does not know which families go forward to be full participants. This includes carrying out observations in the clinics with families who have agreed to be ‘non-participants’, so that the clinic staff do not know which consultations, and therefore which families, are then included in the analysis and writing up of the data. The project obtained ethical approval via the Local Research Ethics Committee (LREC) of the NHS National Research Ethics Service (NRES).

Analysis is based on transcripts of anonymous interviews and the detailed notes of observations. Via independent and then shared coding of transcripts and notes, a theoretically influenced coding frame was developed, which was then applied to the transcripts. Comparative analysis across the transcripts and notes via the coding frame, sat alongside continued engagement with the differing narratives emerging within the individual families (Riessman 1993). The focus for this paper is on the completed analysis of the interviews and observations of all the families who have had their first consultation (the majority of which we observed). The patterns we discuss are those which were evident across the transcripts and observation notes, they are therefore exemplars of the trends in the data.

## The genetic consultation: clues and photographs

Almost all the first consultations we observed involved three key aspects: the close examination of the child’s body, taking photographs of that body, and questions around family history. The geneticists described this process to families as ‘looking for clues’. Much of the process of clue gathering centred on identifying unusual features in the child’s physical characteristics, mediated by ways in which they did or did not look like other family members:

The geneticist, while washing hands, turns to the mother and asks: If you see pictures of his brother at the same age, do they look the same?

Mother: No they’re totally different, different hair, different features. His brother takes after me, while he takes after his dad.

Geneticist: So, if we saw a picture of dad at the same age, would they look the same?

Mother: Yes, the double of each other.

Geneticist, still at sink, speaks to child (Jake): You’ve got a lovely smile!

…

Geneticist comes down onto her knees, at eye-level with Jake, who puts his head down …

At this point the geneticist’s tone of voice is very soft, very slow and gentle, speaking to Jake: Can I see your hand too? Thank you very much.

Still speaking in very gentle tone, the geneticist nods to the mother and asks: And you are seeing Dr [X], and you think his eyes are ok?

While speaking, the focus of the geneticist’s gaze remains on Jake all the time.

Mother: They’re fine, though he has got a lazy eye …

Geneticist stands back up: Can we just pop his top off?

Once the mother removes child’s top, the geneticist asks Jake in very upbeat but still gentle tone: Can I just hold you for one minute?

Geneticist then looks at the mother: It’s just to get a sense of his weight, and his body … You’re quite a floppy boy.

(Observation notes, First Consultation, Keddy Family)

The process of gathering clues places the child’s body under intense scrutiny. The focus of first consultations was not blood tests, or the interpretation of DNA microarray analysis, instead it was the embodied presence of the child. The features mark out the child because, while Jake may look like his father, the focus is on identifying what makes him look different from others in the family and ways in which he feels physically different (‘floppy’). The examination of Jake’s physical distinctiveness is the proxy for what may be different about his internal genetic make up. As such the physical characteristics focused on (his hands, and muscle development) move from being visible but benign variation, to being a marker of potential genetic otherness. His internal genetic difference is marked on his body, a powerful symbolic reference point to what makes him different to others in both his family and the broader community. The use of distinctive physical characteristics in the diagnostic process can emphasise the peculiarity of those characteristics and dissects the child into an object of micro-medical scrutiny:

Mother: She [the daughter] was a bit of meat on the bed. The geneticist was doing their job; I don’t have any resentment about that. But it just became, she became like an object. It was very, it just felt clinical and I didn’t like it. The geneticist was looking at bits of her.

(Interview 1, Rushton Family)

During the consultation, photographs are often taken to record the unusual physical features of the child, captured over time and compared to existing photographs of children said to have the same syndrome. In the process the camera becomes a vehicle for cataloguing difference. The kinds of photographs taken are very different from either the formal or informal photographs that a family would place on their wall, digital photo frame, or desktop screen saver. They are intimate in the way they dissect the child into close ups of the aspects of the body telling to diagnosis. Taking each photograph entails the consultant getting very close to the child with the camera, often on their knees on the floor of the consultation room, taking photographs of fingers, feet, nails, hands, ears, eyes and other features. While a professional photographer will use their skills and tricks of the trade (such as lighting) to minimise ways in which a subject might look different or unusual, here the focus is on targeting the ‘strange’, or ‘abnormal’:

Geneticist asks the child (Owen): Mind if I take a picture of you? … It’s for my friends in the clinic.

The geneticist kneels down in front of Owen, with digital camera ready. I [the researcher] can see the geneticist, but Owen has his back to me, sitting on his father’s knee.

Father speaks to Owen: Don’t pull your face.

Geneticist takes one photograph of the child – a shot straight on, of his face. The geneticist then holds the camera out, and asks Owen: Would you like to see yourself?

The geneticist turns the camera round so Owen can see the picture that has been taken in the viewer.

Geneticist: There you go.

Then, looking at the image, the geneticist turns to the mother: They [Owen’s eyes] are a little wide but not anything to worry about.

(Observation notes, First Consultation, Morgan Family)

The formal power to interpret the significance of the digital image shown to the child and parents remains with the geneticist. It is their medical authority which asserts that, while the eyes are a little far apart, there is nothing to worry about. It is their gaze which appears to hold the child within the scope of normal physical development or outside of it. Looking at the interaction as a whole we can also see ways in which the geneticist seeks to de-objectify and re-humanise the process by introducing practices we would more readily recognise as elements of taking a family photograph. One example of this is the suggestion that it is their ‘friends’ who will see the photograph (as opposed to others in the genetics team who will be involved in discussions around diagnosing the child). However, this crossover into the familiarity of a casual photograph is hard to maintain; the medical gaze comes back to authorise the interpretation of the photograph’s significance, even if the process draws from other social registers. As Latimer *et al.* (2006) argue regarding dysmorphology, the clinician is the skilled reader and interpreter of visual difference and similarity.

## Kinship images and stories of difference

In the context of everyday family life, photographs play very different roles from those in the consultation. Both formal family portraits and informal photographs of holidays, birthdays and other symbolically significant occasions are important family practices, which place the camera at the centre of making kinship connections (Atkinson *et al.* 2001, Bouquet 2001). As Finch (2007: 67) has argued, displays of family life, via photographs, paintings, memorabilia, are ‘the process by which individuals, and groups of individuals, convey to each other and to relevant audiences that certain of their actions do constitute “doing family things” and thereby confirm that these relationships are “family” relationships’. Displays of family cannot be separated from the stories of relationships and boundaries that both the stories and images together sustain (Finch and Mason 2000, Ribbens McCarthy *et al.* 2000). A family story ‘reflects a family’s values, culture, and its collective meanings’ (Kellas 2005: 367). As well as showing who belongs, photographs and stories also operate to mark more subtle gradients of belonging, which provide testimony of tensions and disputes within family ties.

In the following discussion of informal photographs and family histories drawn from one particular family, we can see the significance of existing kinship understandings of inheritance and health in the production of familial relations. These representations situate the child being seen by the genetics service as other within the family, regardless of what emerges from the genetics consultation. At the end of the first consultation the mother showed the consultant a mobile phone image of the sister of the child being examined:

The mother looks through her mobile phone for a photograph of her older daughter without glasses on. She says to the geneticist: You can see they’re [her two daughters] totally different.

…

The father finds a picture of their older daughter looking straight ahead and hands phone to the geneticist, who looks at it, then holds it next to the child (Grace) who is being examined: Hm, well, I think you can see lots of similarities, you can tell they’re sisters, though their head shapes are obviously different …

Mother: The [older] sister takes after me, Grace takes after you [to father].

(Observation Notes, First Consultation, Brown/Jones Family)

Significantly, it is the mother who focuses on highlighting how different the children look (‘they’re different’); while it is the geneticist who emphasises similarities the children share as sisters (‘lots of similarities’). In a subsequent interview with the maternal grandmother the same claim – that Grace ‘took after’ her father – was repeated:

The only thing I can see is that Grace looks like her dad. She’s got my daughter’s small features, but other than that I don’t see anything. Now in my other granddaughter, certain mannerisms, she’s me mother, believe it or not, [laughs] I think oh, she’s me mother, born long after me mam died … But I can’t see anything at all in my granddaughter with learning problems, apart from that she looks like her dad and she’s got, but she’s got her mam’s tiny little features, that’s all I can say about her …’cos she is *him*, which takes us back to, well it must be his side.

(Interview 1, Brown/Jones Family, Maternal Grandmother)

The grandmother suggests one granddaughter belongs to the maternal line through claims to recognise shared characteristics, while she aligns the other granddaughter to the father’s side. In doing so the grandmother seeks to frame the sisters as belonging to different and differently valued, heritages within the broader family. The final point by the grandmother directs us to connections being made by both grandmother and mother. The child, they believe, looks like her father: closer physical similarity equates to closer genetic connection; closer genetic connection equates to the source of her problems being him. In so doing she becomes an other to their family, she is the embodiment of the intrusion of difference via the father’s genetic material.

For all the grandmother’s assertions about the source of the apparent genetic fault, when we did the first interview with her, the clinic had not established either what particular syndrome they could associate with the child’s problems, or its possible source. Indeed it could be either parent or neither. Whatever the outcome of the diagnostic process the referral to genetics is already providing a rhetorical device, which supports well established boundaries produced by her. ‘His’ family will always remain separate. The grandmother can legitimise a dislike for him, via a genetic rhetoric of poor stock. He has introduced a ‘substance’ (Schneider 1968, Carsten 2001) into the kinship line, which is not welcomed. Therefore, the process of genetic investigation is not the producer of otherness here; instead it is well-established moral tales of kinship boundaries.

## Visualising similarity

So far we have concentrated on processes – both within diagnostic exploration and kinship association – which emphasise the ways in which a child is different from others in the family and wider community. However, these same processes can also bring the child back into the fold of family and ‘human acceptability’ (Landsman 2003: 1950). In this section we highlight two common occurrences within diagnostic encounters which reject difference in favour of similarity and belonging.

As before, we begin with visual interpretations within the first consultations:

About half way through the consultation the geneticist examines the child (Connor) by looking at him as he stands in front of his mother just in his underpants.

Geneticist: Actually there’s nothing about him to make me suspect an underlying genetic cause [to his height]. He looks very like you!

Mother laughs.

(Observation notes, First Consultation, Dougherty Family)

In this consultation (and others) genetic difference is rejected by interpreting the child as displaying characteristics associated with his biological kin. Familial resemblance marks both the close bond the child shares with others and secures a sense of normality they can share (Strathern 1992). Both geneticists and family members used recourse to familial similarity to reject that there was anything significantly different about how a child looked, and therefore his or her internal genetic makeup:

Geneticist [to child (Emily)]: Sometimes it can be good to give extra growth hormone, even if the tests show you are making enough, it might still make a difference.

…

Geneticist gets up to wash hands, turns to father: How tall are you sir?

Father: Five [foot] ten [inches]

Geneticist: And Emily’s mum?

Father: She isn’t very tall, and Emily’s sister isn’t very tall either.

Geneticist sits back down and looks at the height chart again. Then looks at Emily, talks very softly: Do you mind if I look at your hands? It may seem like a funny thing, but I’m just looking for clues.

(Observation notes, First Consultation, Nuttall Family)

This discussion came more than halfway through the consultation after earlier questions had focused on the child’s height as a probable indicator of an underlying genetic syndrome. However, the observation here pin points the moment when there was a shift in emphasis as the height of other family members is taken into account. Height then moves from being potentially problematic for the child, to being a possible trait that fits more closely within family normative patterns, something instead she shares with her sister and mother. Kinship is, therefore, reintroduced as a potentially less problematic connection through which to understand the child.

The second route through which geneticists refuted difference was to suggest that a child’s physical characteristics were within the realm of normal developmental variation:

Geneticist [wheeling chair up closer to the child (Emily)]: It’s a chart that plots two lines – this one [points to one running along the top] shows the average height measurement for your age, and this line, with all these dots [hand-written, underneath the first line] is where you were at. So there are the two lines, and we can follow where the dots go along compared to the line of averages. Your line is not too far away from the average, but we can see times when your height slows down for a while. We can also look at your bone age, which is a bit more difficult to explain. This is when we do an x-ray to see if we can predict when you might stop growing. Some children stop growing at twelve or thirteen, others keep on growing until they are sixteen. If that’s the case for you, then you may catch up.

All the time the geneticist talks directly to Emily.

(Observation Notes, First Consultation, Nuttall Family)

Here the geneticist emphasises the child’s every day normality (‘you may catch up’). While the focus of the geneticist’s gaze on the child’s body appears to problematise her, the verbal exchange – including the ways in which the child is spoken to directly – refutes objectification and instead asserts the diversity of child development. In interactions such as these, geneticists validate the human worth of a child whatever genetic variation she or he may or may not have:

Maternal Grandmother: One thing I will say, just the, the last parting shot the geneticist who we saw, I thought it was a really nice thing to say, and very true. They said, ‘just take her home and enjoy her. She’s a little girl.’ Didn’t she?

Mother: Yeah

Maternal Grandmother: ‘Like any other mum … just forget about the syndrome, take her home and enjoy her for what she is.’

(Interview 1, Smith/Henderson Family, Mother, Father, Maternal Grandmother)

The reasons given for rejecting difference in all the examples above are enabled via the uncertainty embedded in dysmorphology. The interpretative aspect of the examination and comparison to others (either other children with known genetic difference or a child’s existing kin) facilitates opportunities to identify characteristics which retain the child within the boundaries of normalcy. This creates a space for both geneticists and family members to look at the child in an expansive way, not confined by an authoritative and non-negotiable template.

## Virtual belonging

In this final section before the conclusion we wish to bring in an aspect of the visual processes embedded in diagnosis which takes place outside the formal clinic environment: familial examination of dysmorphology images on the internet. The interpretation of these images is important because they provide an important reminder that the familial processes of making sense of genetic investigation draw on sources of information which families actively seek, independently of what formal services provide (Schaffer *et al.* 2008, Gunderson 2010). A range of websites exist which catalogue the distinctive physical features associated with genetic syndromes and chromosomal variation. How families engage with these representations provides another space within which dynamics of connection and disconnection are made.

All the families followed up consultations where particular syndromes or patterns of genetic variation were suggested with searches on the internet. The original aim was often to find out further information, in particular what kind of future their child could expect. What quickly caught their attention were the images they found, and how their child did or did not look like them. The Smith/Henderson Family discussed the range of images they found, stressing the feelings of unease and then reassurance they had experienced. The first sites found by the parents contained images of children older than their daughter (Sophie), but with a range of very marked physical abnormalities (alongside cognitive variation too). They had been clearly troubled by how physically different the children looked (they were particularly alarmed by pictures of children who looked like ‘dwarfs’). The potential inference that their child may look like that in the future, and therefore less like them, was deeply upsetting. Eventually the maternal grandmother found an alternative site, which offered an alternative future for the child which was not as marked by difference. On the site she found photographs of an older child, which also contained pictures of her at a similar age to her granddaughter. The grandmother quickly shared them with the mother and father:

Father: There is also, when you look into it, some recent case histories with other people, sort of adults, where this is one, there’s one little girl, when they show you a picture of her when she was first born, and she is identical to Sophie.

Maternal grandmother: That’s what I saw, I showed [mother], I says, I got quite a shock when I saw her.

Father: and then you see a picture of her when she’s four and

Maternal grandmother: She’s beautiful isn’t she?

Father: And it was completely different

Mother: Yeah she’s lovely, she is a normal little girl, she’s just little.

(Interview 1, Smith/Henderson Family, Mother, Father, Maternal Grandmother)

These pictures of an older child offered an alternative future for their daughter/grand-daughter, which the family could positively imagine and incorporate into their version of family and connection. The internet site listed a range of possible traits (both physical and cognitive) associated with the syndrome. Because the child they saw looked most like the children who also appeared to develop fewer cognitive problems, the key message they took was that their daughter/granddaughter would also be less affected by such problems. Sophie was, therefore, both closer to their reading of ‘normal’ and, by implication, to being recognised as part of the family.

The context within which the next family interpreted the images they saw on the internet provides an interesting insight to how – at least some – families may respond to the kinds of genetic information produced about chromosomal deletions and translocations made possible by DNA microarray analysis in the future. The Todd/Richardson Family were informed via telephone prior to a second consultation that the analysis of their son’s blood tests (in this instance using fluorescence in situ hybridisation [FISH], a forerunner to microarray analysis) appeared to show that he had a particular deletion on a numbered chromosome. Soon after the parents received this information, they were on the internet and found images of other children who had what they believed to be the same deletion. They were immediately struck by how similar their child looked to the other children, confirming for them that their child must have that deletion. When discussing this with the researcher before the consultation, the father said that when he looked at the images he thought he was looking at his own child. It was therefore a considerable surprise to be told in the consultation that what the geneticists had found was a specific form of deletion pattern on the numbered chromosome which they would not have seen on the internet:

Mother: we’ve been on the internet and seen lots of things, and some pictures. We can see a lot of Harry in them.

…

Geneticist starts to explain the chromosome results.

…

Geneticist [pointing to page]: You’ll see things named this on the internet, with Chromosome [N]. But what Harry has is Chromosome [N] point [N], which is different to what you mostly see on the internet.

…

What Harry has is [pointing to sections of his drawing of chromosomes] one copy of chromosome [N] which is completely normal, and one completely normal but with a tiny bit missing. This can cause a whole series of different things to happen.

…

We see about half a dozen kids a year with chromosome [N point N] deletion, and the one thing that is true is that they all vary a bit between them. This is difficult for you, as parents, because we can’t say exactly how things will be for each child. But it’s important to say it doesn’t change Harry. He is still Harry. So this is really just giving us an explanation about things going on for him.

(Observation notes, Second consultation, Todd/Richardson Family)

When the father reflected later he argued that it didn’t matter that his son’s deletion was different to those children whose images he had originally seen:

Father: We could see how all these children look the same. Just having something different about your genes brings them all together, whatever the deletion-point-this-that-or-the-other is.

(Observation notes, Second consultation, Todd/Richardson Family)

While the analysis of his son’s blood is able to produce a level of detail about the specific pattern of deletion, it carries little meaning or value for the father. As the consultant acknowledges, it provides little additional knowledge about what the future holds. Instead, what was meaningful for both the mother and father was seeing other children whose specific pattern of chromosomal variation may be different, but who look similar. The specific chromosome deletion does not in itself define the child – as different or similar. It is a piece of technical information, which says so little about the child now or in the future, that it cannot become a factor in framing him. Instead, the child becomes defined for the parents by images of children who look similar, found on the internet.

On the websites cataloguing dysmorphic features said to be associated with specific syndromes or chromosomal variation, children’s images are placed within a specific form of recognition and belonging. Being able to see that other children exist who carry a similar mark of genetic difference in their physical features can support processes of ‘watchful waiting’ parents undertake as they try to imagine futures for their children. Families find it troubling to see ‘grotesque’ versions of the characteristics associated with a syndrome. In contrast images of ‘normal looking’ children who they imagine to look like their child can produce feelings of comfort and security. The image – and therefore their child – remains within the category of the ‘acceptably human’ (Landsman 2003).

## Conclusion

The stories and images brought to the foreground during genetic investigation of children with a possible rare and complex genetic variation have the potential to place those children in the category of the other, or reclaim them as valued members of kinship relations, and through this also as legitimate members of society. It is hard to deny the way in which the detailed examination of a child’s body within a consultation frames her or him as problematic. The authority of the medical gaze has the power to define a child’s features as problematic. However, that is different from saying the child is positioned as problematic. For a variety of reasons this outcome is often escaped. First, the full dynamics of the consultation, via speech as well as bodily movement, include deliberate reminders of the child’s normalcy and the variation embedded in all children’s development. Second, there is a space within both dysmorphology and chromosomal analyses for those around the child to continue to see her or him as similar to them, as one of them. This space occurs through the ambiguity which lies at the heart of the visual interpretation of the child’s body. Craft expertise remains central to diagnosis and is a form of expertise which remains open to negotiation and fluidity of meaning. Uncertainty is also present when such analyses and interpretations move into the realm of future prognosis. Due to the rareness and complexity of the genetic variations being potentially identified, the geneticists struggle to say anything definitive about what the future holds. While parents find that uncertainty frustrating, it is the presence of uncertainty throughout the diagnostic process which allows them, and others, to exercise their own judgment based on their position within kinship worlds. It is the uncertainty contained within the diagnostic processes which enables all concerned to creatively engage with the child in such a way that escapes ‘fixing’ her or him into the category of the ‘genetically other’.

What this discussion of our study has also shown is how framings of difference as other often already exist around the child prior to potential genetic explanations entering the picture. This is because the developmental problems the child has require explanation. This requirement is in order to get support for the child, to help imagine the future and also, at times, to apportion or counter blame. Such demands for explanation emerge from within existing and culturally common familial stories of the past and present, within which family boundaries and senses of belonging are formed.

The final aspect to note is the need to maintain the distinction between difference and other. A child can be thought of as developmentally different or genetically different and still be thought of as belonging to a family, to society. It is important to highlight the ways in which geneticists and family members are able to recognise the child as different and still ‘one of us’. Genetic exploration does not close off the possibility that difference in either how the child looks, or the child’s capabilities, is understood as simply part of the spectrum of human variation and distinctiveness. Recognition of connection and value is not solely dependent on being seen as the same as others. But one word of caution, quite often in the acknowledgements by geneticists or by family members that a child was different, but still okay, was a focus on minimising that difference. The implication is that the other remains present within images of other children or futures in genetics’ websites, which parents do not want to imagine as possible scenarios for their child. The sense of relief that both geneticists and family members expressed when they interpreted a child as not belonging to those other children who were ‘grotesque’ implies that they would have struggled to incorporate significant difference in development and appearance into either their sense of humanness or of family. Of course we know such incorporation exists within families who love and cherish children who are significantly different. What the expressions of tangible relief witnessed in our study point to is that the – apparently – similar and normal child is easier to place within the boundaries of what it is to be human and what it is to be a kinship member.

## Footnotes

1 To capture a retrospective perspective, the second stage of the fieldwork aims to do in-depth interviews with families (including parents, siblings, significant family and friends, and the children themselves) who have at least 5 years’ break from being last seen by the paediatric genetic service.
